# Supplementary material for: The Systems Biology Research Tool: evolvable open-source software
Source: BMC Syst Biol. 2008 Jun 29;2:55. doi: 10.1186/1752-0509-2-55 (PMC2446383; doi:10.1186/1752-0509-2-55)
Supplement: Additional file 1 — SBRT Archive. An archive of the current version of the Systems Biology Research Tool. [file 1752-0509-2-55-S1.zip › sbrt-1.4.0/doc/developers_guide/api/sbrt/kernel/fba/class-use/ExchangeRxn.html]

Uses of Class sbrt.kernel.fba.ExchangeRxn


|  |  |  |
| --- | --- | --- |
| **Overview** \| **Package** \| **Class** \| **Use** \| **Tree** \| **Deprecated** \| **Index** \| **Help** | |  |
| PREV   NEXT | **FRAMES**    **NO FRAMES**     **All Classes** |  |


---


## **Uses of Class sbrt.kernel.fba.ExchangeRxn**

| Packages that use ExchangeRxn | |
| --- | --- |
| **sbrt.kernel.fba** | Provides classes and interfaces for creating objects relevant to Flux Balance Analysis. |

Uses of ExchangeRxn in sbrt.kernel.fba

| Subclasses of ExchangeRxn in sbrt.kernel.fba | |
| --- | --- |
| `class` | `SinkRxn`             This class is used to represent sink reactions in Flux Balance Analysis. |
| `class` | `SourceRxn`             This class is used to represent chemical species sources in Flux Balance Analysis. |

| Methods in sbrt.kernel.fba that return ExchangeRxn | |
| --- | --- |
| `ExchangeRxn` | `IrrevRxnFluxomeV1.getExchangeRxn(java.lang.String reactionName)`             Returns the exchange reaction in this fluxome with the provided name. |

| Methods in sbrt.kernel.fba that return types with arguments of type ExchangeRxn | |
| --- | --- |
| `java.util.Map<java.lang.String,? extends ExchangeRxn>` | `Fluxome.getExchangeRxns()`             Returns the set of all exchange reactions contained in this fluxome. |
| `java.util.Map<java.lang.String,ExchangeRxn>` | `IrrevRxnFluxomeV1.getExchangeRxns()`             Returns all of the exchange reactions contained in this fluxome. |

---


|  |  |  |
| --- | --- | --- |
| **Overview** \| **Package** \| **Class** \| **Use** \| **Tree** \| **Deprecated** \| **Index** \| **Help** | |  |
| PREV   NEXT | **FRAMES**    **NO FRAMES**     **All Classes** |  |


---
